# Merkel Cell Polyomavirus Large T Antigen Unique Domain Regulates Its Own Protein Stability and Cell Growth

**DOI:** 10.3390/v12091043

**Published:** 2020-09-18

**Authors:** Nnenna Nwogu, Luz E. Ortiz, Hyun Jin Kwun

**Affiliations:** 1 Department of Microbiology and Immunology, Pennsylvania State University College of Medicine, Hershey, PA 17033, USA; nxn5200@psu.edu (N.N.); lzg271@psu.edu (L.E.O.); 2 Penn State Cancer Institute, Hershey, PA 17033, USA

**Keywords:** Merkel cell carcinoma, skin cancer, Merkel cell polyomavirus, Large T antigen, cell proliferation, E3 ligases, protein stability, viral latency, MCV LT unique regions

## Abstract

Merkel cell polyomavirus (MCV) is the only known human oncogenic virus in the polyomaviridae family and the etiological agent of most Merkel cell carcinomas (MCC). MCC is an aggressive and highly metastatic skin cancer with a propensity for recurrence and poor prognosis. Large tumor antigen (LT), is an essential oncoprotein for MCV transcription, viral replication, and cancer cell proliferation. MCV LT is a short-lived protein that encodes a unique domain: MCV LT unique regions (MURs). These domains consist of phosphorylation sites that interact with multiple E3 ligases, thus limiting LT expression and consequently, viral replication. In this study, we show that MURs are necessary for regulating LT stability via multiple E3 ligase interactions, resulting in cell growth arrest. While expression of wild-type MCV LT induced a decrease in cellular proliferation, deletion of the MUR domains resulted in increased LT stability and cell proliferation. Conversely, addition of MURs to SV40 LT propagated E3 ligase interactions, which in turn, reduced SV40 LT stability and decreased cell growth activity. Our results demonstrate that compared to other human polyomaviruses (HPyVs), MCV LT has evolved to acquire the MUR domains that are essential for MCV LT autoregulation, potentially leading to viral latency and MCC.

## 1. Introduction

Merkel cell carcinoma (MCC) is a rare but highly aggressive cutaneous neuroendocrine carcinoma [1]. Approximately 80% of Merkel cell carcinomas are caused by Merkel cell polyomavirus (MCV or MCPyV), the only known oncogenic human polyomavirus (HPyV) [2]. MCV is ~5.3 kilobase (kb) viral genome that encodes an early gene locus involved in viral replication, and a late gene locus encoding capsid structure proteins. An early region transcript, MCV large T (LT) protein is generated as a result of alternative splicing of the T antigen locus of the early region [3]. MCV LT is a highly phosphorylated protein that recognizes its viral DNA replication origin and forms a double-hexameric helicase complex to initiate replication of the viral genome [4,5]. 

MCV LT is a spliced transcript comprising 2 exons producing a protein 817 amino acids in size [3]. The N-terminal end of MCV LT (1–70 aa) contains the DnaJ domain encoding the specific HPDKGG sequence responsible for Hsc70 binding (42–47 aa) [4] and a conserved LXCXE motif for retinoblastoma (Rb)-binding which has high homology to other polyomaviruses [3]. Similar to other polyomaviruses, MCV LT antigen contains multiple conserved domains including a nuclear localization signal (NLS) [6], the origin-binding domain (OBD) [4], and the carboxyl terminal (C-terminus) half which contains several critical elements essential for DNA binding, helicase activity and viral replication [4,5]. In contrast to Simian virus 40 (SV40) LT, expression of wild-type MCV LT inhibits cell proliferation [7,8]. Further analysis of the MCV genome clonally integrated into MCV-positive tumor cells revealed mutations that induce premature truncations in the LT antigen [3], resulting in the deletion of the C-terminus growth-inhibitory domain [7,8]. 

Protein phosphorylation is a reversible posttranslational modification essential for regulating protein function [9]. Previous reports have shown that phosphorylation is a regulatory mechanism associated with T antigen regulation in Murine polyomavirus (MPyV) and SV40, known polyomavirus models [10,11,12]. The phosphorylation of SV40 LT and MPyV LT is essential for regulating LT antigen function, specifically, viral replication [10,13,14]. MCV LT is a phosphoprotein and contains approximately 82 potential phosphorylation sites (p-sites), which are expected to be crucial for modulation of LT protein function [15]. Full-length LT initiates viral replication and induces Ataxia telangiectasia mutated (ATM) and Ataxia-telangiectasia- and Rad3-related (ATR)-mediated DNA damage response (DDR) and cell cycle arrest, leading to cell death [8,16,17]. It was previously observed that MCV LT serine (S) 816 phosphorylation by the ATM kinase stimulates DDR and cell growth inhibition [17]. Interestingly, an alanine (A) substitutional mutation of S816 only partly restores cell growth, suggesting the possibility of a secondary mechanism contributing to this underlying phenotype [17]. 

Growing evidence indicates that regulation of viral protein stability may be a key determinant for viral pathogenesis and oncogenesis in many human tumor viruses [18,19,20,21]. SV40 LT protein is relatively stable (t1/2 > 36 h), and regulated at lysine (K) 697 by a reversible acetylation reaction [22]. The effect of acetylation on SV40 LT stability is not changed by proteasomal inhibitor, MG132, indicating that lysine 697 acetylation has a predominant role in regulating SV40 LT stability. Moreover, Shimazu et al. described that a substitution mutation of K679 to glutamic acid (Q) reduced SV40 LT stability and cell growth, suggesting that LT stability is closely related to LT-mediated viral and host protein–protein interactions and cell proliferation. 

MCV LT contains a unique domain that exhibits no homology to other polyomaviruses [23,24]. This region comprises two fragments: MCV unique region (MUR) 1 (106 aa) and MUR2 (39 aa), which flank the Rb binding motif [23,24]. This domain contains a unique vacuolar sorting protein (Vam6p) binding sequence (W209) [23], and phosphorylated serines (S147, S220, S239) that interact with Skp-Cullin-F-box (SCF) E3 ligases [15]. MCV LT interactions with SCF E3 ligase complexes induce self-destabilization [15], thus, we hypothesized that this domain functions as a negative regulatory element for LT-mediated cell growth. We sought to determine whether MCV LT MUR domain affects LT protein-mediated cell proliferation. Our results show that deletion of the MUR in MCV LT significantly increases MCV LT protein stability and cell proliferation due to loss of SCF E3 ligase interactions. In contrast, the insertion of MCV LT MUR sequences to SV40 LT destabilizes SV40 LT protein by enhancing SCF E3 ligase interactions, resulting in a moderate decrease in cell proliferation. Interestingly, upon deletion of the MUR domain, MCV LT retained its ability to replicate MCV viral origin and regulate LT-mediated MCV early gene transcription. Our results reveal that MCV LT MUR domain contributes to the regulation of LT stability and host cell proliferation, which may explain a critical interplay evolved in MCV, leading to human cancer. 

## 2. Materials and Methods

### 2.1. Cell Culture and Transfection

BJ-hTERT and 293 cells were cultured in Dulbecco’s modified Eagle’s medium (DMEM) with 10% premium grade fetal bovine serum (FBS) (Seradigm, Providence, UT, USA). Transfections with expression vectors were performed using jetOPTIMUS (Polyplus) according to the manufacturer’s instructions. For stable BJ-hTERT LT-expressing cells, lentivirus-transduced cells were selected with 3 µg/mL puromycin (Sigma-Aldrich, St. Louis, MO, USA) for two days. Because either insertion or deletion of the MUR in LT resulted in reduced expression levels of LT mutants due to changes in stability and stochastic dynamics, the optimal amount of mutant DNA used for transfection was adjusted to obtain comparable protein expression in each experiment.

### 2.2. Plasmids

Codon-optimized, commercially synthesized MCV LT antigen sequences were cloned into pcDNA6/V5/His vector (Invitrogen, California, CA, USA) with a modified multiple cloning site (MCS) (Addgene 40200) [25]. All LT mutations were generated by overlapping PCR mutagenesis. MCV LT S147A, S220A, S239A mutants were previously described [15]. MCV LT S142A mutant was generated using primer pair (5′-GTC GAG CGC GTC GGG GTA CGG GAG CTT CTC AG-3′, 5′-CTG AGA AGC TCC CGT ACC CCG ACG CGC TCG AC-3′). To construct SV40 LT expression plasmid, SV40 LT cDNA sequence (Addgene 9053) [26] was cloned into the same vector used for MCV LT expression using primer pairs (5′-CCG CGA TAT CAC CAT GGA TAA AGT TTT AAA C-3′, 5′-CTG GGC CCT CGA GTT ATG TTT CAG G-3′). The MCV replication origin plasmid (Ori339(97)) [4], MCV transcription reporter (pRL/Ori/FL) [15], HA-FBW7ΔDF(d231-324) [27], HA-β-TrCP, HA-Skp2, and Flag-cMyc [15] have been previously described. For lentiviral constructs, pLVX-eGFP-P2A empty vector was constructed by overlapping PCR using primers (5′-GAG CGA TAT CAC CAT GTA TCC CTA TGA CG-3′, 5′-GAA GTT AGT AGC TCC GCT TCC GGT GAT ACC GGC AG-3′). All LT sequences were cloned into the pLVX-eGFP-P2A empty vector using *Afe*I and *Bam*HI restriction enzyme sites. The following primers were used for the MUR mutations of LTs: MCV LT dMUR1 (5′-GGG GGA TTT TCG TTT ACT TGG GAA GAT CTT TTC TGC GAC G-3′, 5′-GAA AAG ATC TTC CCA AGT AAA CGA AAA TCC CCC-3′); MCV LT dMUR2 (5′-CTG CGA CGA ATC GCT GTC AGA GGA GTA CCG CAG CTC-3′, 5′-GAG CTG CGG TAC TCC TCT GAC AGC GAT TCG TCG CAG); SV40 LT+MUR (5′-GCA GTG GTG GAA TGC CTT TGG TAA AGC CTA TGA GTA TGG-3′, 5′-CCA TAC TCA TAG GCT TTA CCA AAG GCA TTC CAC CAC TGC-3′, 5′-CCC AGT TTA CAG ACA CTC CTC CAA AAA AG-3′, 5′-CTT TTT TGG AGG AGT GTC TGT AAA CTG GG-3′).

### 2.3. Merkel Cell Polyomavirus Large Tumor Antigen (MCV LT) Domain Analysis

Sliding window plot (6 aa window size) of 14 HPyVs LT amino acid sequence similarities (DQ305492.1, NC_001699.1, NC_009238.1, GU296381.1, JF813003.1, HM011563.1, HM011564.1, NC_014361.1, NC_015150.1, JQ898292.1, NC_020106.1, NC_020890.1, NC_024118.1, NC_034253.1) were compared using PLOTCON (EMBOSS). For disordered region prediction, IUPred2 and ANCHOR2 were utilized to identify disordered protein regions and disordered binding regions of MCV LT, respectively.

### 2.4. Cell Proliferation Assay

BJ-hTERT (2.5 × 10^3^ cells/well) cells expressing LTs were seeded in 96-well plates. A Cell Counting Kit-8 (Sigma) was used for quantitation of viable cell count in proliferation assay at each time point according to the manufacturer’s instructions. A cell proliferation assay was performed in triplicate in the presence of 0.1% fetal bovine serum (FBS). 

### 2.5. MCV Origin Replication by Quantitative Polymerase Chain Reaction (qPCR) Analysis

The MCV replication origin assay has been previously described [4]. 293 cells were transfected with an expression vector (LT) and pMCV-Ori339(97) using Lipofectamine 2000 or 3000 (Invitrogen, California, CA, USA) in 12-well plates. Episomal DNA was collected by salt-precipitation at 48 h post transfection; 1 µg of DNA was digested with *Dpn*I, then 50 ng of digested DNA was subjected to quantitative polymerase chain reaction (qPCR). qPCR was carried out with PowerUp^TM^ SYBR Green Master Mix (Applied Biosystems, Carlsbad, CA, USA) using a StepOnePlus^TM^ system (Applied Biosystems) according to the manufacturer’s protocol. Primer sequences used for MCV origin detection were previously described [4]. 

### 2.6. MCV Transcription Analysis

A dual bidirectional reporter construct was used to measure MCV gene transcription [15]. The MCV promoter/origin region was cloned into a bidirectional dual Firefly (early) and Renilla (late) luciferase reporter (pRL/Ori/FL) [15]. The activity of the Firefly (early gene reporter) and Renilla (late gene reporter) luciferase were determined sequentially using the dual-luciferase reporter assay (Promega, Madison, WI, USA) according to the manufacturer’s protocol. A plasmid expressing eGFP [28] was used as an internal transfection efficiency control. eGFP protein expression was analyzed using a Synergy 2 fluorescence reader (Biotek, Winooski, VT, USA). The levels of the experimental reporter activity were normalized to the eGFP fluorescence intensity.

### 2.7. Quantitative Immunoblotting and Antibodies

LT protein turnover was measured by a cycloheximide (CHX) chase assay using quantitative immunoblot analysis. LT plasmids were co-transfected with an eGFP plasmid to normalize transfection efficiency, and all experiments were performed in triplicate. Cells were lysed in IP buffer (50 mM Tris-HCl (pH 8.0), 150 mM NaCl, 1% TritonX-100, 1 mM PMSF, 1 mM benzamidine) and whole cell lysates without pre-clearing were used for direct immunoblotting. Primary antibodies were incubated overnight at 4 °C, followed by 1 h of secondary antibody incubation at room temperature. All signals were detected using quantitative infrared (IR) secondary antibodies (IRDye 800CW goat anti-mouse, 800CW goat anti-rabbit, 680LT goat anti-rabbit IgG, 680LT goat anti-mouse IgG) (LI-COR); signal intensities were analyzed using a laser-scanning imaging system, Odyssey CLX (LI-COR). CM2B4 (Santa Cruz Biotechnology, Dallas, TX, USA), 2T2 (Millipore, Billerica, MA, USA), PAb416 (Santa Cruz Biotechnology), c-Myc (9E10, Santa Cruz Biotechnology), GFP (D5.1, Cell Signaling) HA-Tag (C29F4, Cell Signaling, Danvers, MA, USA), anti-FLAG (M2, Sigma-Aldrich, St. Louis, MO, USA), anti-α-Tubulin (12G10, DSHB), β-Actin (13E5, Cell Signaling) antibodies were used for this study.

### 2.8. Lentiviral Transduction

For lentivirus production, 293FT (Invitrogen) cells were used for induction according to the manufacturer’s instructions. Lentivirus infection was performed in the presence of 6 µg/mL polybrene (Sigma-Aldrich). BJ-hTERT cells were selected with puromycin (3 µg/mL) after lentivirus transduction with pLVX-eGFP-P2A empty vector, wild-type LTs (MCV LT WT, SV40 LT WT) and LT MUR mutants (MCV LT dMUR, SV40 LT+MUR).

### 2.9. Flow Cytometric In Situ Proximity Ligation Assay

Proximity ligation assay (PLA) was performed using the Duolink assay kit (Sigma-Aldrich, St. Louis, MO, USA) according to the manufacturer’s instructions. To evaluate LTs and SCF E3 ubiquitin ligase interactions, LTs were co-expressed with HA-FBW7ΔDF(d231-324) [27] or HA-Skp2 [15] or HA-β-TrCP [15]. Primary antibodies were utilized at optimized concentrations with HA-Tag (C29F4) (1:500), 2T2 (1:500) (Millipore) and SV40 T antigen (PAb416) (1:250) (Santa Cruz Biotechnology). Cells were analyzed by flow cytometry on a 16-color BD LSR Fortessa. The acquired data was analyzed using FlowJo software (Tree Star, Ashland, OR, USA).

### 2.10. Immunofluorescence

BJ-hTERT cells were grown on glass coverslips. After 48 h, cells were fixed with 2% paraformaldehyde in PBS, washed 3 times with wash buffer (0.3 M glycine in phosphate-buffered saline (PBS)), and permeabilized in 0.3% Triton X-100 for 10 min. The cells were washed 3 times with wash buffer and blocked by preincubation with 0.3 M glycine in 5% bovine serum albumin (BSA) for 1 h at 37 °C. Cells were labeled with the appropriate primary antibodies and then incubated with the appropriate Alexa Fluor-conjugated secondary antibody. Cells were analyzed with a REVOLVE4 fluorescent microscope (Echo Laboratories). 

## 3. Results

### 3.1. MCV LT Has a Unique and Disordered Domain Structure

**Figure 1:**
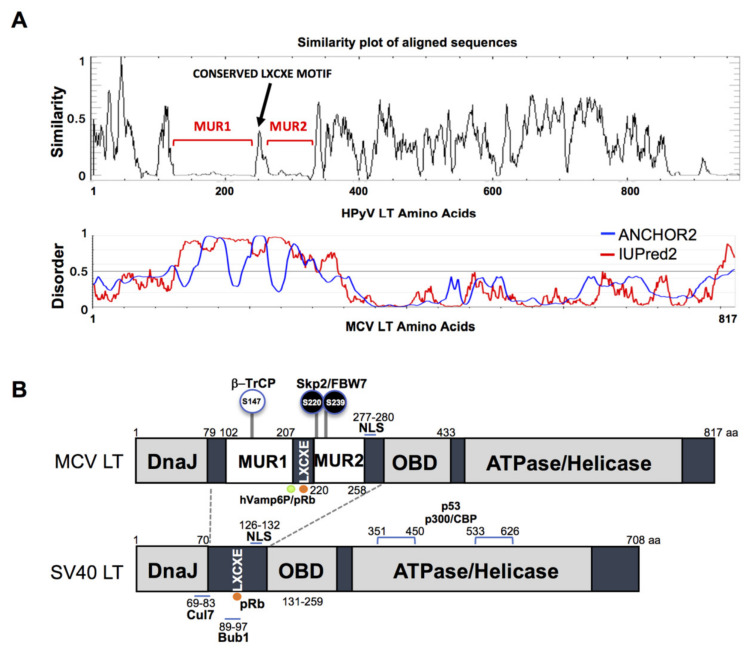
Merkel cell polyomavirus large tumor antigen (MCV LT) similarity and disorder plots. (**A**) LT amino acid similarity of 14 human polyomaviruses (HPyVs) and disordered region of MCV LT protein. Sliding window plot (6 aa window size) analysis. LT amino acid similarity was compared using PLOTCON (EMBOSS). For disordered region prediction, IUPred2 and ANCHOR2 were utilized to identify disordered protein regions and disordered binding regions in MCV LT, respectively. MCV LT contains a unique disordered region (MUR) divided in two fragments by the conserved LXCXE motif [23,24]. (**B**) Diagram of MCV LT and SV40 LT domain structures. Compared with SV40 LT, MCV LT has an extended structure, MUR, that serves as an interacting domain with multiple cellular factors. The MUR domain also consists of phosphorylated serines for SCF E3 ligase recognition (phospho-degron motifs) [15].

The gene sequence of MCV LT is highly conserved among all MCV strains harboring an intact LT sequence [3]. We compared the aligned LT amino acid sequences of 14 human polyomaviruses using PLOTCON software (EMBOSS). As previously shown, the Rb-binding motif in MCV LT is located between the two unique regions (MUR1 and MUR2), unlike other polyomaviruses (Figure 1A) [23,24]. Further assessment of these sequences using IUPred2 and ANCHOR2 allowed us to investigate these MUR fragments, and we ascertained that these two regions are intrinsically disordered (IUPred2 analysis), which may contribute to the multi-functionality of LT including protein-protein interactions [29,30,31]. Because SV40 LT protein interactions and functions are well characterized, we compared sequence variations between SV40 LT and MCV LT (Figure 1B). 

### 3.2. MCV LT Unique Region (MUR) Is Important for LT Stability

**Figure 2:**
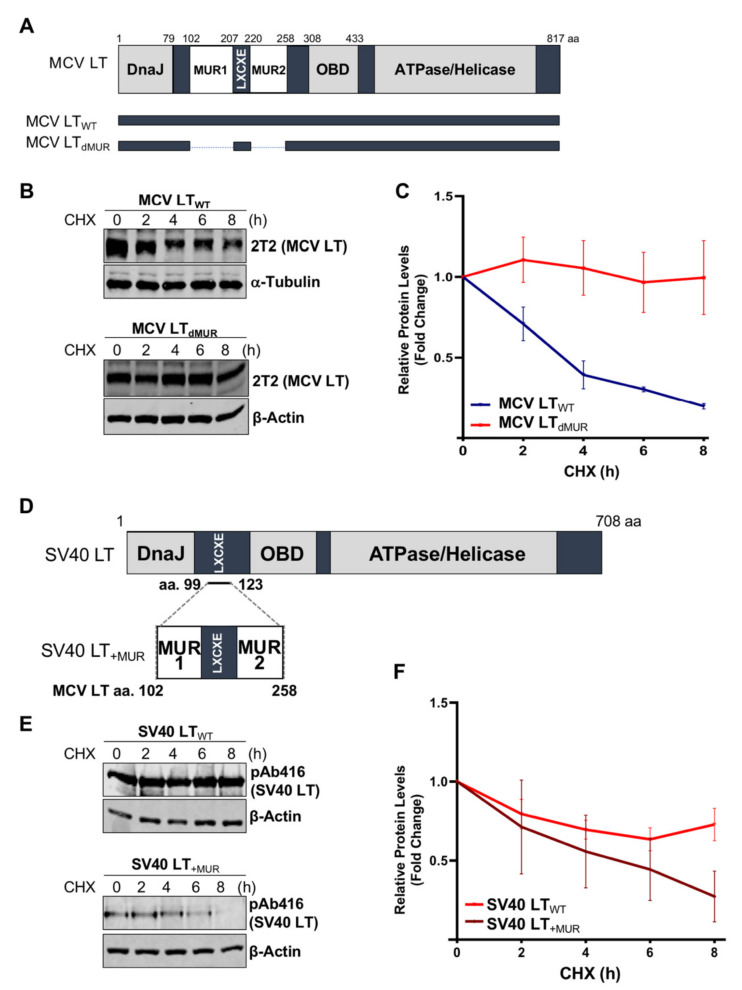
MCV LT unique region (MUR) regulates LT stability. (**A**) Diagram of MCV LT coding regions and sites of deletion mutations. MCV LT MUR domains: MUR1 (102–207 aa) and MUR2 (220–258 aa) were simultaneously deleted to evaluate LT stability. (**B**) LT MUR destabilizes LT. LT protein turnover was measured by a cycloheximide (CHX) chase assay using quantitative immunoblot analysis. Cells transfected with LT_WT_ or LT_dMUR_ constructs (0.3 µg and 0.9 µg respectively) were treated with CHX (0.1 mg/mL) 24 h after transfection and harvested at each time point indicated. (**C**) Protein expression was quantified using a LI-COR IR imaging system. Deletion of the MUR extended the half-life of LT from ~3–4 h up to >8 h. Error bars represent SEM and were calculated using GraphPad Prism software. Data were analyzed using three biological replicates per experiment, *n* = 3. (**D**) Diagram of SV40 LT coding regions and sites of insertion mutations. SV40 LT amino acids (99–123) were deleted, and the MCV LT complete MUR domain (102–258 aa) was inserted. (**E**) MCV LT MUR destabilizes SV40 LT. SV40 LT protein turnover was assessed by a CHX chase assay using quantitative immunoblot analysis. 293 cells transfected with either wild-type SV40 LT (SV40 LT_WT_) (0.3 µg) or SV40 LT_+MUR_ (0.6 µg) were treated with CHX (0.1 mg/mL) 24 h after transfection and harvested at each time point indicated. (**F**) Protein expression was quantified using the laser-scanning Odyssey CLX (LI-COR) infrared (IR) imaging system. MCV MUR insertion into SV40 LT reduced SV40 LT turnover to ~6 h. Error bars represent standard errors of the mean (SEM) and were calculated using GraphPad Prism software. Data were analyzed using three biological replicates per experiment, *n* = 3.

Previous studies determined that MCV LT MUR domains have multiple E3 ligase binding serines that are not present in SV40 LT [15,32]. To understand the effect of the MUR domains on MCV LT stability, we created a LT MUR deletion mutant (LT_dMUR_) (Figure 2A) and LT protein stability was analyzed in the presence and absence of the MUR domain using a CHX chase assay. As shown in Figure 2B,C, deletion of the MUR domain significantly reduced LT protein turnover. To further verify if this stability phenotype was MUR-specific, we introduced the MUR sequences to SV40 LT (SV40 LT_+MUR_) (Figure 2D). Our analysis illustrated that introduction of the MUR mutation to SV40 LT significantly reduced protein stability as shown in Figure 2E. Immunoblot analysis demonstrated a significant reduction in SV40 LT half-life of 36 h [22] to approximately 6 h (Figure 2F). 

### 3.3. Functional Analysis of the MUR in MCV Transcription and Replication

**Figure 3:**
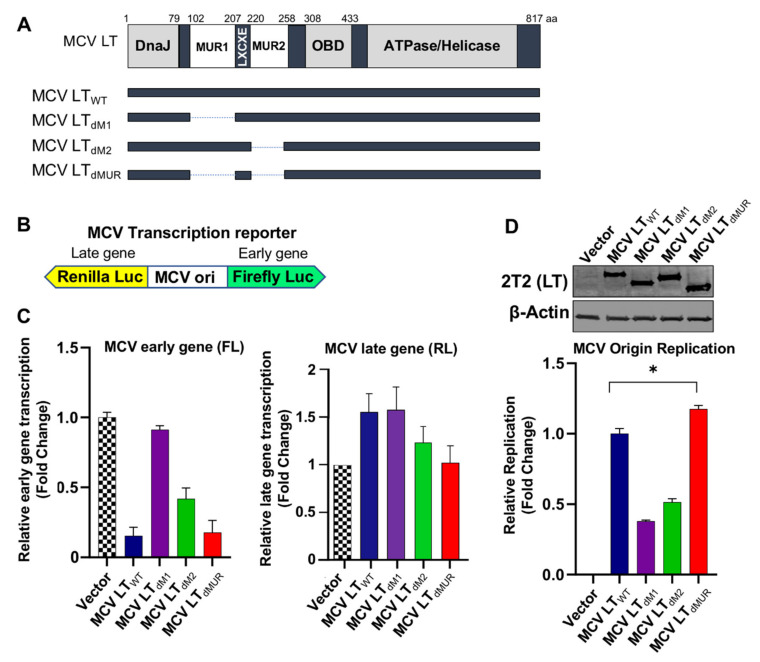
MCV LT maintains origin binding and replication capacity independent of the MUR domain. (**A**) Schematic of MCV LT coding regions and sites of deletion mutations. MCV LT MUR1 (102–207 aa) (LT_dM1_), MUR2 (220-258 aa) (LT_dM2_) or MUR1+MUR2 (LT_dMUR_) (102-258aa) were deleted to evaluate LT mediated viral replication. (**B**) Diagram of MCV transcription reporter. The MCV promoter region (nt 4928–195; GenBank accession no. EU375804) was cloned into a bidirectional dual Firefly (early) and Renilla (late) luciferase reporter [15]. (**C**) Early gene transcription activity was measured by luciferase activity using co-transfection of the reporter (0.5 µg) with either wild-type LT or MUR deletion mutants (0.5 µg) into 293 cells. Relative luciferase activity was normalized to empty vector control (mean ± SEM, *n* = 3). Deletion of either MUR1 or MUR2 impaired repression of MCV early gene transcription while deletion of both MUR1 and MUR2 (dMUR) successfully downregulated MCV early gene transcription compared to wild-type LT. There was no significant increased late gene transcription by either LT_wt_ or mutant LTs because no robust amplification of origin replication occurred as shown in Figure 3D. Similar results are observed using the replication-deficient reporter [15]. (**D**) MCV origin replication assay. Either deletion of MUR1 or MUR2 impaired MCV origin replication. Deletion of both MUR1 and MUR2 (LT_dMUR_) retained its ability to replicate MCV origin as compared to wild-type LT. Error bars represent SEM and were calculated using GraphPad Prism software. Data were analyzed using three biological replicates per experiment, *n* = 3. Differences between means (*, *p* value ≤ 0.05) were analyzed using a *t*-test with GraphPad Prism software.

Next, we sought to determine if MCV LT MUR affected LT ability to regulate MCV viral origin replication and transcription. To evaluate the functions of the MUR domain on MCV viral transcription, we constructed a series of LT MUR deletion mutants (LT_dM1_, LT_dM2_) (Figure 3A). To evaluate the functions of wild-type LT (LT_WT_) and LT MUR mutants (LT_dM1_, LT_dM2,_ and LT_dMUR_) on MCV viral transcription, we used a bidirectional reporter containing the MCV non-coding control region (NCCR), where the origin of replication (ori) is located (Figure 3B) [15]. The bidirectional reporter was then co-transfected with LT-expressing plasmids, and simultaneous expression of early gene was determined. The binding of wild-type and mutant MCV LTs to the ori was evaluated by measuring early gene transcription activity where an increase in luciferase activity signified failed binding of MCV LT to the ori and, conversely, lower luciferase activity inferred efficient binding of MCV LT to the ori [4,15]. We observed that LT_WT_ significantly repressed MCV early gene transcription, as previously reported [15]. In contrast, the deletion of either MUR1 or MUR2 resulted in a decreased ability of LT to inhibit early gene transcription (Figure 3C). Moreover, the deletion of either MUR1 or MUR2 significantly reduced the LT capacity to replicate the MCV origin, mostly due to changes in DNA binding properties (Figure 3D). Surprisingly, the double MUR deletion mutant, LT_dMUR_, permitted efficient binding of MCV LT to the ori at a similar level as LT_WT_, which, in turn, restored the LT function to regulate MCV early gene transcription and viral replication (Figure 3C,D). No significant changes in late gene transcription by expression of either LT_WT_ or LT mutants were observed as previously reported [15], suggesting a substantial role for the MUR structure in efficient lytic viral replication.

### 3.4. MCV LT MUR Is Critical for Cell Growth Inhibition

**Figure 4:**
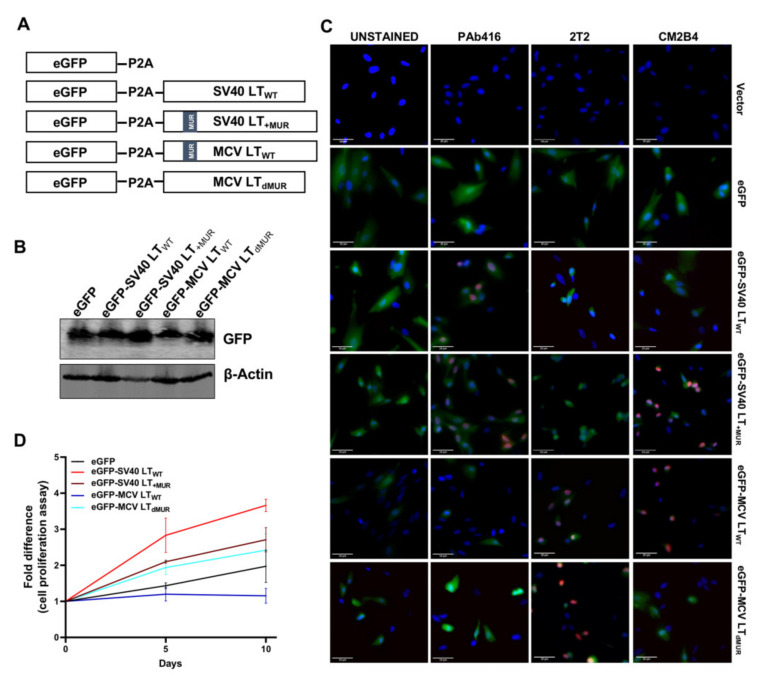
MCV LT MUR regulates cell growth inhibition activity of LT. (**A**) Diagram of MCV LT and SV40 LT lentiviral constructs used for cell proliferation analysis. Enhanced GFP (eGFP) was tagged to the N-terminus. P2A self-cleaving peptide was fused between eGFP and LT sequences to allow non-tagged LT expression. (**B**) Immunoblot analysis of eGFP expression in BJ-hTERT fibroblasts. eGFP can effectively be used as a marker of LT expression. (**C**) Validation of the P2A lentivirus system by immunofluorescence staining. Cells were fixed, and GFP fluorescence was analyzed by direct visualization, whereas LT antigen expression was identified by indirect immunofluorescence using specific antibodies. Nuclear counterstain (DAPI-Blue), GFP for T antigen expression (Green), and various T antigen antibodies (Pab416, 2T2, CM2B4) (Red). Scale bar = 10 µm. (**D**) MCV LT MUR regulates serum-independent human BJ-hTERT cell proliferation. LT proteins were transduced by lentiviral vector into immortalized BJ-hTERT cells, and cell proliferation was determined in 0.1% FBS using a CCK-8 colorimetric cell proliferation assay. Wild-type LT mediated cell growth inhibition, as previously reported [7,8]. In the absence of MUR, LT accelerated BJ-hTERT cell growth (normalized by mean OD values on day 1 for two independent experiments performed in triplicate). In contrast, wild-type SV40 LT promoted cell proliferation while the insertion of MCV MUR decreased cell proliferation potential in the absence of serum.

To evaluate the effect of LT turnover on cell growth, we assessed cell proliferation rate in hTERT-immortalized BJ human foreskin fibroblasts (BJ-hTERT) transduced with lentivirus expressing wild-type LT (LT_WT_) and LT MUR deletion mutants (LT_dM1_, LT_dM2,_ and LT_dMUR_). A lentiviral eGFP-LT gene expression system using 2A self-cleaving peptide (P2A) derived from porcine teschovirus-1 [33] was created for the wild-type and mutant plasmids (Figure 4A). When P2A successfully mediates the simultaneous expression of LT and cleavage of the eGFP fusion gene, eGFP can function as a marker for LT expression (Figure 4B).

To further validate the efficacy of our P2A cleavage system and to detect LT protein, we performed immunofluorescence staining using LT-specific antibodies (PAb416, 2T2, CM2B4). While PAb416 recognizes an epitope within amino acids 83–128 of the SV40 LT [34] and 2T2 from the DnaJ domain of MCV LT [35], CM2B4 was raised against amino acids 116–129 (RSRKPSSNASRGA) located within the MUR domain of MCV LT [36]; thus, CM2B4 LT antibody specifically recognizes proteins containing the MUR domain. As expected, SV40 LT and MCV LT were detected in the nucleus by specific antibodies, PAb416 and 2T2 respectively. CM2B4 recognized both SV40 LT_+MUR_ and MCV LT_WT_, while SV40 LT_WT_ and MCV LT_dMUR_ were undetectable. (Figure 4C). 

BJ-hTERT fibroblasts cells expressing MCV LT_WT_ grew at a rate slower than that of the vector control cells, as previously reported [7,8]. This growth inhibitory effect was significantly ablated by the deletion of the MUR domain, indicating that LT stability contributes to LT-mediated cell growth (Figure 4D). Although cells stably expressing SV40 LT_WT_ and SV40LT_+MUR_ grew faster than cells expressing the eGFP vector control, SV40LT_+MUR_ expression partially decreased cell growth rate in comparison to SV40 LT_WT_ (Figure 4D). Summarily, our results demonstrate that the MUR domain plays a critical role in MCV LT stability and, consequently, regulates cell proliferation.

### 3.5. MCV LT Has Evolved to Acquire the MUR Domain for LT Autoregulation

Efficient analysis of the interaction between E3 ligases and their target substrates can be very challenging. This is due to the weak and transient physical interaction, as well as rapid dissociation between some E3 ligase-substrate complexes [37]. The predicted p-sites associated with F-box and WD repeat domain-containing 7 (FBW7), S-Phase Kinase Associated Protein 2 (Skp2), and Beta-transducin repeats-containing proteins (β-TrCP) recognition are located within the MUR domain [15]. Although MCV LT and E3 ligase interactions have been characterized by in vitro co-immunoprecipitation, these assessments are often plagued with extensive troubleshooting. Moreover, these in vitro techniques do not identify whether this interaction occurs endogenously, therefore, they may not reflect the native behavior of their counterparts. Consequently, we utilized an in situ PLA analysis combined with flow cytometry to revalidate LT interaction with FBW7, Skp2 and β-TrCP E3 ligases. Previously described degron sites for FBW7 (S239), Skp2 (S220), and β-TrCP (S142 and S147) [15] were further evaluated to confirm these interactions. We also assessed the interaction between these E3 ligases and SV40 LT_+MUR_ to verify our hypothesis.

**Figure 5:**
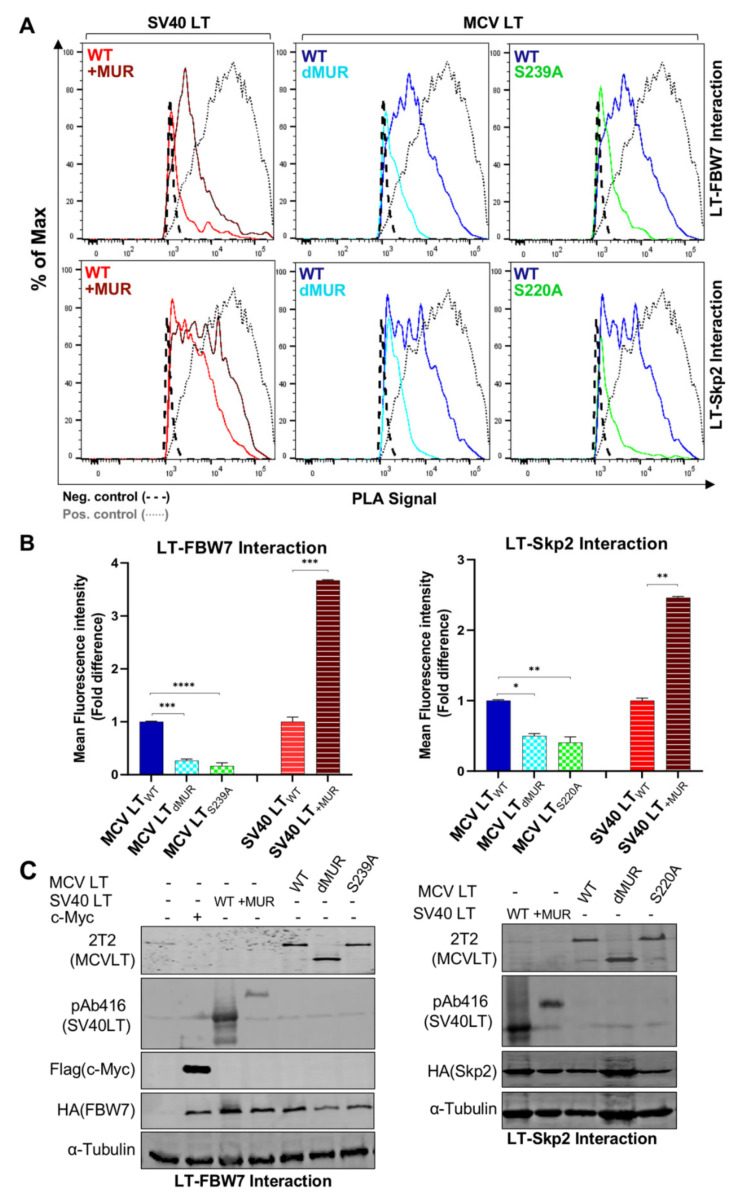
MCV LT MUR is LT-FBW7 and Skp2 interaction domain. (**A**) MCV LT MUR interacts with SCF E3 ligase complexes. HA-tagged F-box proteins (FBW7 and Skp2) (6 µg) were co-transfected with LTs (6 µg) into 293 cells, and proximity ligation assay (PLA)-flow cytometric analysis was performed with anti-HA and either 2T2 (MCV LT) or pAb416 (SV40 LT) antibodies. c-Myc/FBW7 interaction was included as a positive control for PLA-flow cytometry. Wild-type LT mediated E3 ligase interaction through the known serine phosphorylation sites within the MUR domain, as previously reported [15]. Insertion of the MCV LT MUR into SV40 LT (SV40 LT_+MUR_) increased Skp-Cullin-F-box (SCF) protein interactions. (**B**) Calculated fold difference of mean fluorescence intensity of PLA analyzed by Flow Jo software. (**C**) Protein expression was evaluated by immunoblot analysis.

**Figure 6:**
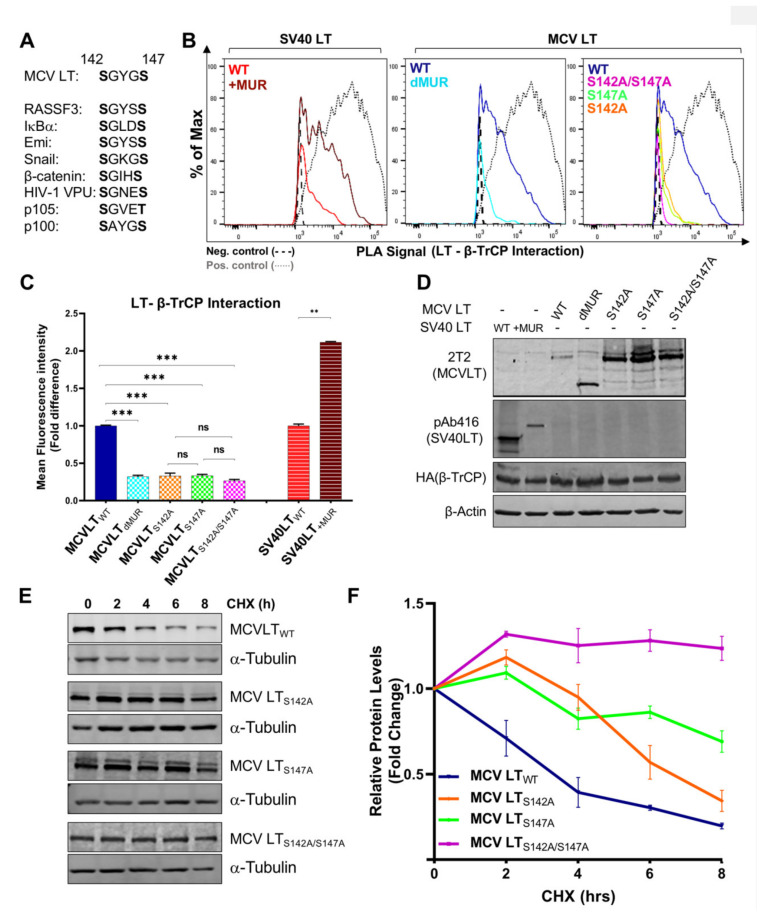
Serines 142 and 147 are the LT phosphorylation sites for β-TrCP recruitment. (**A**) β-TrCP binds to the DpSGXX(X)pS dual-site phosphorylation motif in its substrates [38]. (**B**) MCV LT MUR interacts with β-TrCP. β-TrCP (6 µg) was co-transfected with LTs (6 µg) into 293 cells, and PLA-flow cytometric analysis was performed with anti-HA and either 2T2 (MCV LT) or pAb416 (SV40 LT) antibodies. c-Myc/FBW7 interaction was included as a positive control for PLA-flow cytometry. (**C**) Calculated fold difference of mean fluorescence intensity of PLA analyzed by Flow Jo software. (**D**) Protein expression was evaluated by immunoblot analysis. (**E**) LT alanine (Ala, A) substitution mutants at S142 and S147 potential β-TrCP binding residues were tested for stability by a CHX chase assay using quantitative immunoblot analysis. Cell transfected with LTs (0.5 µg) were treated with CHX (0.1 mg/mL) 24 h after transfection and harvested at each time point indicated. (**F**) Protein expression was quantified in triplicate using an LI-COR IR imaging system. Error bars represent SEM; n = 3. Both S147 and S142 to Ala mutations along with the double mutant increased LT stability. The double mutant is the most stable of all three while S147A mutant is more stable than S142A.

Consistent with the previous reports, our results showed that full-length MCV LT_WT_ efficiently interacts with FBW7 [15,27,32] and Skp2 [15,19] and these interactions are impeded by the deletion of the MUR domain (Figure 5A). Quantification of MCV LT_dMUR_ interaction with both FBW7 and Skp2 resulted in a significantly low-intensity PLA signal in comparison to MCV LT_WT_ under comparable protein expression conditions (Figure 5B). These interactions were also markedly diminished by the introduction of alanine mutations at their specific phospho-degron recognition sites S239 and S220, respectively, under comparable protein expression conditions (Figure 5A–C), consistent with previous reports [15,19]. Our PLA analysis further validated these serine sites as crucial phosphorylation sites for LT-E3 ligase interactions. Conversely, the addition of the MUR domain to SV40 LT significantly increased SV40 LT binding to these E3 ligases, corroborating our hypothesis that the MUR domain is critical for these interactions. We also evaluated the binding modality of β-TrCP and MCV LT. The S142-S147 sequence in MCV LT has been previously described as a canonical β-TrCP degron site (Figure 6A) located within the MUR domain [15]. Assessment of MCV LT_dMUR_ interaction with β-TrCP yielded a significantly low-intensity PLA signal in comparison to MCV LT_WT_ under comparable protein expression conditions and, conversely, insertion of the MUR domain to SV40 LT increased SV40 LT interaction with β-TrCP (Figure 6B). F-box protein substrate binding requires specific phosphorylation of the target substrates, and β-TrCP binding requirement entails the phosphorylation of serine residues at a consensus sequence, DpSGXXpS, as seen in most β-TrCP substrates [38]. Due to this double phosphorylation requirement, we further examined and replicated the interaction study of S142 and S147 alanine substitution mutants (MCV LT_S142A_ and MCV LT_S147A_) with β-TrCP along with a double mutant S142A/S147A (MCV LT_S142A/S147A_). Both single and double alanine mutations of S142 and S147 resulted in a significant loss of LT interaction with β-TrCP at comparable protein levels (Figure 6B–D), suggesting that at least one of phosphorylation events is required for LT binding to β-TrCP. We further analyzed the effect of the alanine mutations on LT stability determined by a CHX chase assay. Our results showed a significant increase in LT stability upon mutation at S142 and S147 degron sites (Figure 6E,F). Furthermore, our double mutant S142A/S147A displayed an even higher level of stability in comparison to the single mutants.

## 4. Discussion

Studies have shown that rapidly evolving disordered regions are key functional domains and play essential roles in modulating cell signaling pathways [29,30,39]. These intrinsically disordered regions (IDRs) are critical regulators of numerous cellular mechanisms and are frequently linked to posttranslational modifications [40], and other transient protein interactions [41]. Their state of structural disorder permits a flexible and dynamic structure, making them susceptible to phosphorylation [42,43,44]. Intrinsically disordered proteins (IDPs) have been linked to multiple malignancies, and approximately 70% of human cancer-associated proteins have been predicted to retain disordered regions [45,46]. Moreover, intrinsic disorders have been observed in crucial oncoproteins of multiple human oncogenic viruses, including human papillomavirus (HPV) [47,48], human T lymphotropic virus type 1 (HTLV-1) [49] and Kaposi’s sarcoma-associated herpesvirus (KSHV) [50]. 

MCV LT is a multifunctional oncoprotein that plays vital roles in the viral life cycle and tumorigenesis. Here we show that the MUR sequence is uniquely able to inhibit cell growth by destabilizing LT protein via multiple SCF E3 ligase interactions at known serine phosphorylation sites. There are multiple regulatory mechanisms involved in the mediation of E3 ligase substrate recognition, including posttranslational modifications (PTMs) such as sumoylation, ubiquitylation, and phosphorylation [51,52]. These PTMs are very abundant in highly disordered regions [40,43,45], thus making them attractive binding targets for E3 ligase interactions. In the case of Mdm2 [53], Gli3 [54], Sic1 [55,56], cyclin E [57], and Mcl1 [58], phosphorylation at several sites are necessary before their putative SCF enzyme recognize the protein [59,60]. FBW7 and Skp2 target cell cycle regulators and modulate cell cycle progression, cell proliferation, and apoptosis, key hallmarks of tumorigenesis. The identification of substrates for the SCF complex has proven difficult, especially given the requirement of multiple phosphorylation events allowing for a switch-like recognition, ubiquitination, and degradation modality [55,61]. There is a paucity of information on the binding modality of E3 ligases and although the presence of SCF phospho-degron motifs has been used to separate true substrates from non-specific interactors, E3 ligase substrates often do not use its optimal consensus motifs (or canonical sequence) [55,62,63,64]. There are known FBW7 substrates that do not have the canonical CPD consensus sequence (pS/pTPXXpS/pT) including but not limited to mTOR T631 (TPSIH) [65], PGC-1 alpha T263 (TPESP) [66], TP63 S383 (SVSQL) [67], and Sic1 (TPSTP, TPQKP, SPFNG, SPFPK, TPSDK [55] for Cdc4 binding in yeast models. Nonetheless, substrate recognition and degradation by either FBW7 or Skp2 require phosphorylation reactions within their specific degron motifs [62,68,69,70] and a sequential multi-phosphorylation cascade of LT may be involved in E3 ligase recognition. These various interaction modes illustrate the tunability of the E3 ligase system for substrate binding. 

Substrate recognition by β-TrCP mainly occurs in a dual phosphorylation-dependent manner. These substrates have a relatively conserved degron site that contains DpSGXXpS or analogous variants [71] which includes serine residues that must be phosphorylated for successful β-TrCP recognition [72]. β-TrCP substrates such as β-catenin, and Snail are phosphorylated at their degrons after an initial priming phosphorylation event that permits binding to a secondary phosphorylation site [62,73,74,75]. Single serine mutations within the canonical β-TrCP degron site S142-S147 significantly diminished β-TrCP-MCV LT interaction. Furthermore, the double mutation at the degron sites inhibited β-TrCP-MCV LT interaction to similar levels as the single mutations inferring that mutation of either serine 142 or 147 is sufficient to inhibit β-TrCP-MCV LT interaction. This was further validated by observation of S142 and S147 mutations inducing an increase in LT stability. Moreover, in numerous β-TrCP substrates, the degron sites are surrounded by additional p-sites which facilitate the phosphorylation of the two serine residues present within the β-TrCP degron [76]. Because S147 in LT, but not S142, was identified as a phosphorylation site [77], the initial mutation study focused on the S147 [15]. This in vitro p-site mapping study may suggest that the phosphorylation event on S147 appears to make the predominant contribution to LT and β-TrCP binding in physiological conditions. Nonetheless, our results demonstrate that both phosphorylation events are involved in β-TrCP binding and may be required for viral replication [15]. 

HPyVs are commonly found in the human population. While HPyVs generally do not produce any noticeable symptoms, several HPyVs are associated with human diseases. Although a possible oncogenic role for BK polyomavirus (BKV) remains controversial in human cancers, it has shown an association of BKV infection in renal carcinogenesis, implying that diverse oncogenic mechanisms may apply [78]. Polyomaviruses have a unique gene expression profile that is entirely dependent on viral replication and life cycle. Our study focused on full-length MCV LT and interactions that may occur during the early events of viral infection. Further studies are necessary to elucidate the interplay between viral oncogenes and cellular molecules in the polyomavirus life cycle. 

## 5. Conclusions

MCC is the most lethal skin cancer. Despite substantial progress made in the elucidation of MCC tumorigenesis, it is currently unclear how cellular and environmental co-factors are involved in MCV pathogenesis and MCC development. Our study demonstrates a functional role of MCV LT unique region by which MCV LT uniquely influences LT stability and cellular proliferation, potentially a key feature leading to the development of MCC.
